# Where do Eritrean migrants get infected with malaria? The importance of considering the migration route

**DOI:** 10.2807/1560-7917.ES.2019.24.6.1900095

**Published:** 2019-02-07

**Authors:** Philippe Gautret, Martin P Grobusch, Patricia Schlagenhauf

**Affiliations:** 1 University Hospital Institute for Infectious and Tropical Diseases, Aix-Marseille University, Marseille, France; 2 Centre for Tropical Medicine and Travel Medicine, Department of Infectious Diseases, Amsterdam University Medical Centers, University of Amsterdam, Amsterdam, The Netherlands; 3 WHO Collaborating Centre for Travel Medicine, Travel Clinic and Department of Public Health, Epidemiology, Biostatistics and Prevention Institute, University of Zürich, Zürich, Switzerland

**Dear Editor**: We read with interest the paper by Sonden et al. [1] confirming the large increase in numbers of *Plasmodium vivax* cases diagnosed in Europe during the years 2014 and 2015, mainly due to newly arrived Eritrean migrants. Using the GeoSentinel surveillance network data, we recently published similar trends with regards to *P. vivax* in Eritrean migrants presenting in Europe but also in Israel [2]. Among 146 Eritrean migrants with malaria presenting at GeoSentinel clinics between 1999 through September 2017, the proportion of *P. vivax* infections was 84.2% (123/146), followed by *P. falciparum* (12/146; 8.2%), which does not reflect the local epidemiology in Eritrea, where there is a large predominance of *P. falciparum* reported [3,4]. A further point of interest was finding four cases of *P. ovale* (4/146; 2.7%) and one case of *P. malariae* (1/146, 0.7%) when the proportion of these two species is 0.02% of all malaria cases in studies conducted in Eritrea [4]. The remaining six cases were either mixed *P. vivax/P. falciparum* infections or unknown species. Among 100 patients with information available, 69 (69%) presented with symptoms after arrival and the median time between arrival in the host country and presentation was 39 days; 31/100 (31%) of migrants reported malaria symptoms while in transit. Among *P. vivax* infections, we also reported 4% (5/123) cases of severe *P. vivax* malaria.

This situation raises several important questions – where do the Eritrean migrants get infected with malaria on their migration route? Our study [2] proposed that the acquisition of *P. vivax* likely occurred somewhere underway, possibly in Sudan or Ethiopia, or perhaps even in camps in Libya, a country that is currently considered malaria-free; this question is of medical and epidemiological importance. Competent malaria vectors are present in Libya and as camps are populated with migrants, some of whom may be infected by *Plasmodium* spp. including *P. vivax*, the possibility that malaria may be transmitted in such camps should be considered – and investigated by entomological surveys, despite the challenge that it may represent.

Early diagnosis and correct management of malaria in Eritrean migrants is necessary to avoid severe disease and the potential reintroduction of malaria in receptive European areas. Primaquine treatment i.e. 15(−30) mg base per day for 14 days, is indicated to prevent relapses by hypnozoite elimination; it is considered standard of care to test for G6PD deficiency in all persons before use of primaquine. We conducted a survey among GeoSentinel clinics regarding availability of primaquine and G6PDH deficiency testing. Our results were that in several European countries, primaquine is only available through hospital pharmacies or through an international pharmacy with delays in procurement and results of testing may take days (1-14 days) [2]. Point of care G6PDd tests are largely unavailable. The procurement logistics and long therapy duration with primaquine as well as the need for pre-treatment G6PD screening in a potentially ‘hard-to-reach’ population call for a specific policy to address malaria in Eritrean migrants.

Based on published literature [5], the infectious diseases most frequently reported in Eritrean migrants (besides malaria) are louse-borne relapsing fever, scabies with secondary bacterial infections, tuberculosis and schistosomiasis. Such infections (except schistosomiasis) likely result from the overcrowding, poor hygienic conditions, exposure to arthropods and risks for transmission of tuberculosis that are typically experienced by migrants during their long migration process. Thus, screening for infectious diseases and differential diagnosis in both asymptomatic and ill migrants should take in account both the endemicity of diseases in the origin country and the risks faced along the migration routes and in holding camps.

## References

[1] Sondén K Rolling T Wångdahl A Ydring E Vygen-Bonnet S Kobbe R Malaria in Eritrean migrants newly arrived in seven European countries, 2011 to 2016. Euro Surveill. 2019;24(5):1800139. 10.2807/1560-7917.ES.2019.24.5.1800139 30722809 PMC6386211

[2] Schlagenhauf P Grobusch MP Hamer DH Asgeirsson H Jensenius M Eperon G Area of exposure and treatment challenges of malaria in Eritrean migrants: a GeoSentinel analysis. Malar J. 2018;17(1):443. 10.1186/s12936-018-2586-9 30497487 PMC6267801

[3] Sintasath DM Ghebremeskel T Lynch M Kleinau E Bretas G Shililu J Malaria prevalence and associated risk factors in Eritrea. Am J Trop Med Hyg. 2005;72(6):682-7. 10.4269/ajtmh.2005.72.682 15964950

[4] World Health organisation (WHO). World Malaria Report 2017. Geneva: World Health Organization; 2017. Available from: https://www.who.int/malaria/publications/world-malaria-report-2017/en/

[5] Isenring E Fehr J Gültekin N Schlagenhauf P Infectious disease profiles of Syrian and Eritrean migrants presenting in Europe: A systematic review. Travel Med Infect Dis. 2018;25:65-76. 10.1016/j.tmaid.2018.04.014 29702253

